# Natural History of Adrenal Steroidogenesis in Autoimmune Addison’s Disease Following Diagnosis and Treatment

**DOI:** 10.1210/clinem/dgaa187

**Published:** 2020-04-17

**Authors:** Catherine Napier, Kathleen Allinson, Earn H Gan, Anna L Mitchell, Lorna C Gilligan, Angela E Taylor, Wiebke Arlt, Simon H S Pearce

**Affiliations:** 1 Institute of Genetic Medicine, International Centre for Life, Newcastle University, Newcastle upon Tyne; 2 Newcastle upon Tyne Hospitals NHS Trust, Royal Victoria Infirmary, UK; 3 Institute of Metabolism and Systems Research (IMSR), University of Birmingham, Birmingham, UK; 4 NIHR Birmingham Biomedical Research Centre, University Hospitals Birmingham NHS Foundation Trust and University of Birmingham, Birmingham, UK

## Abstract

**Context:**

The natural history of adrenal function in autoimmune Addison disease once diagnosed and treated has not been systematically studied, but several case reports of recovery from established adrenal failure suggest it may not be uniform.

**Objective:**

To ascertain steroidogenic function in autoimmune Addison disease immediately following diagnosis and during prolonged treatment.

**Design:**

We studied peak serum cortisol in response to ACTH_1-24_ in 20 newly diagnosed autoimmune Addison disease patients at first presentation and then again within a month. We also studied 37 patients with established Addison disease (for between 7 months and 44 years) in a medication-free state, measuring peak serum cortisol responses to ACTH_1-24_ and the urine LC-MS steroid metabolome.

**Results:**

Adrenal steroidogenesis declined rapidly after steroid replacement treatment for newly diagnosed Addison disease was started, with a peak serum cortisol falling from 138 ± 19 nmol/L (SEM) at presentation to 63 ± 13 nmol/L over 4 weeks (*P* < 0.003).

Six of 37 participants (16%) with established Addison disease had detectable serum cortisol and urine glucocorticoid and mineralocorticoid metabolites during repeat testing, indicating variable degrees of residual adrenal function.

**Conclusion:**

Autoimmune Addison disease is a heterogeneous condition, showing a rapid decline in adrenal steroidogenesis during the first few weeks following diagnosis, but low-level residual function in a minority of patients, which appears to persist for many years.

Autoimmune Addison disease (AAD) is a rare endocrinopathy characterized by immune-mediated destruction of the adrenal cortex (1). Without treatment, it is invariably fatal, but once diagnosed health can be maintained with daily glucocorticoid and mineralocorticoid replacement tablets (2-4). Symptoms of AAD frequently start months or years before the condition is diagnosed (5, 6), and current thinking is that it progresses to a “point of no return” where complete adrenocortical steroidogenic failure is inevitable and universal (7). Despite this assumption, several instances showing apparent spontaneous remission of well-characterized AAD have been reported in patients several years after an initial firm diagnosis (8-10). In addition, 1 study reported reduction in adrenal autoantibody titer and improved adrenal function in a presymptomatic individual following high-dose prednisolone therapy for coexisting Graves’ orbitopathy (11). These studies start to suggest that adrenocortical steroidogenesis may exhibit some plasticity both at first presentation of Addison disease, and perhaps persisting postdiagnosis and treatment of AAD. With this in mind, we previously studied 13 patients with established AAD and found that 2 (15%) had detectable steroidogenesis 4 and 8 years postdiagnosis, which was amenable to improvement with high-dose ACTH therapy (12). One of these individuals remains healthy without steroid medication more than 7 years later, although ACTH remains elevated and adrenal autoantibodies remain detectable.

Whether there is residual adrenocortical steroidogenesis in some AAD patients has therefore become an important issue, because it likely indicates preservation of adrenal capsular stem cells (13-15), which are able to continuously repopulate the adrenal cortex despite autoimmune attack. This provides a potential therapeutic window, whereby persisting adrenal progenitor or stem cells may be harnessed to regenerate adrenal function. However, studies looking at residual steroidogenesis in AAD patients have come to differing conclusions. First, a pioneering study from Utrecht studied 27 AAD patients using a conventional cortisol immunoassay and found detectable ACTH_1-24_-stimulated serum cortisol in 10 patients, but concluded that there was no useful recovery of adrenal function in any subject (16). In contrast, a smaller but more recent study found detectable serum 11-deoxycortisol in 10 of 20 AAD patients using a more sensitive liquid chromatography (LC) tandem mass spectometry (MS/MS)-based steroid assay (17), suggesting a prevalence of residual adrenal function of around 50%. Both studies must be interpreted with the proviso that participants took dexamethasone (16) or hydrocortisone (17), respectively, before steroid measurements, which may have either suppressed ACTH, leading to an underestimation of steroidogenic capacity, or cross-reacted with the cortisol assays in the case of hydrocortisone. In this current study, we present data concerning the very early natural history of AAD during the first few weeks of conventional treatment and revisit the prevalence of residual adrenal steroidogenesis in a larger cohort of established AAD patients. We measured ACTH_1-24_-stimulated serum cortisol in all patients and carried out comprehensive urine steroid metabolite profiling by LC-MS/MS.

## Patients and Methods

### Newly presenting autoimmune Addison disease

In 20 patients, serum cortisol data were captured at first presentation with newly diagnosed Addison disease, followed by a plasma ACTH measurement and a formal short Synacthen (ACTH_1-24_ stimulation) test within 4 weeks of the commencement of oral glucocorticoid replacement. These participants were recruited for enrollment into 2 different studies (NCT00753597, ISRCTN20220821) that attempted to improve adrenal function early on in the natural history of the condition (18, 19). In both studies, participants were diagnosed with Addison disease in local National Health Service hospitals or at our tertiary center and started on oral hydrocortisone replacement therapy. Serum cortisol measurements were captured from the day of their presentation and compared with the peak serum cortisol following Synacthen stimulation 14 to 28 days later when they were assessed during a 36-hour medication-free baseline visit for the intervention study (18, 19). All participants had plasma ACTH concentrations above the reference range before testing and all but 1 patient had positive serum steroid 21-hydroxylase antibodies; this patient went on to develop Graves’ disease, indicating a likely autoimmune basis for her adrenal failure, too.

### Established Addison disease

Thirty-seven patients with established AAD (diagnosis established more than 6 months before study participation) were recruited from the endocrine clinic at our center (Newcastle upon Tyne Hospitals National Health Service Trust). A diagnosis of AAD was confirmed by the subjects having either a low basal cortisol level with a high ACTH level or a subnormal response to the short Synacthen test (250 µg parenteral synthetic ACTH_1-24_). Patients with primary adrenal failure resulting from adrenal gland infiltration or infection, with secondary adrenal failure, or with autoimmune polyglandular syndrome type 1 were excluded (20). Following written, informed consent, they omitted their regular glucocorticoid and mineralocorticoid medication for a 24-hour period, leading to a 36- to 40-hour medication-free window. One participant (patient B) was previously tested during 2012 (reported as patient 06 in reference 12). Detailed safety information was given to the participants to postpone their participation if they had intercurrent illness, along with direct access to a member of the medical team throughout the steroid-free interval. At 18:00 on the steroid-free day, participants started an overnight (14-hour) urine collection for LC-MS/MS analysis of the steroid metabolome. The following morning, between 08:00 and 09:00, the urine collection was stopped, baseline blood was taken for plasma ACTH and serum steroid hormone measurements, and a short Synacthen test using 250 µg of IM ACTH1-24 (tetracosactide) was performed, with repeat cortisol measurements at 30 and 60 minutes. Following the Synacthen test participants were restarted on their usual medications. All studies were performed following National Research Ethics Service approval with identification numbers 08/H0903/32, 12/NE/0339, and 15/NE/0312.

### Steroid measurements

Serum cortisol was measured by competitive chemiluminescent assay (Roche I), lower limit of detection 20 nmol/L. A healthy peak cortisol response to ACTH_1-24_ on this assay is a serum cortisol ≥ 550 nmol/L. Plasma ACTH was measured on a solid-phase, chemiluminescent assay, lower limit of detection = 5 ng/L, reference range 10 to 47 ng/L. Urine steroids were hydrolyzed and extracted by solid phase extraction as described previously (21) and analyzed by LC-MS/MS at the Institute of Metabolism and Systems Research at the University of Birmingham, UK (22). To assess assay precision, pooled urine samples from 6 healthy males were extracted 4 times with the study samples; for all steroids, a relative standard deviation of less than 10% was observed (0.9%-6.6%). Accuracy was assessed at 3 concentrations, 20, 400, and 1000 ng/mL, and ranged from -19.6% to 21.4% (excluding 5PD, PD, and THB as < lower limit of quantitation), -18.9 to -1.2%, and -2.4 to 14.3% at low, medium, and high concentrations, respectively.

### Statistical analysis

Comparison of parametric continuous data (age, steroid doses) was performed using unpaired Student *t*-test. Categorical data were analyzed by Fisher exact test. Nonparametric continuous data (duration of disease, serum ACTH, cortisol measurements) were analyzed by Mann-Whitney *U* test. Correlation between serum cortisol and urinary steroid metabolites was performed using a Spearman rank test. All analysis was performed using Prism v0.8.2.0 software.

## Results

### Newly presenting Addison disease

**Figure 1.**
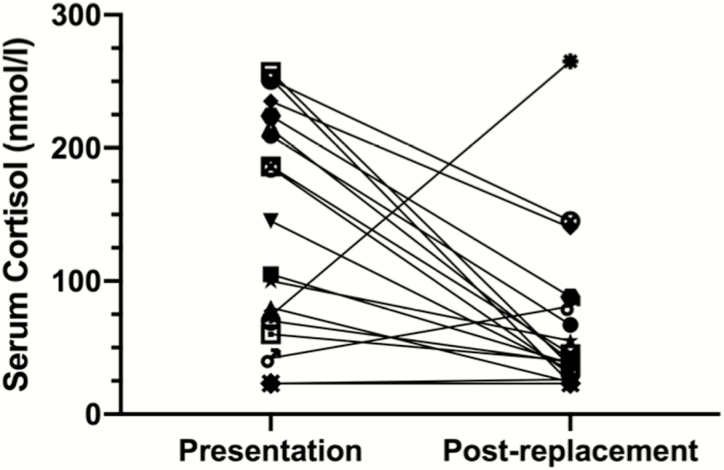
Newly diagnosed Addison disease. Peak serum cortisol at presentation with autoimmune Addison disease and at 1 month postdiagnosis. Serum cortisol concentration measured at first presentation of Addison disease is shown in the left-hand column linked to the cortisol concentration taken a median of 26 days later in the same individual in the right-hand column. A normal peak cortisol response on this assay is ≥ 550 nmol/L. The presentation serum cortisol fell from a median of 125 nmol/L (range < 23-257) to 39.5 nmol/L (range < 23-265), (*P* < 0.007; Mann-Whitney *U* test). To convert serum cortisol concentrations from nmol/L to μg/dL, divide by 27.6.

Twenty newly presenting AAD patients (age range, 17–64 years, 13 females) were studied on the day of first presentation with adrenal insufficiency, either with random or ACTH_1-24_–stimulated serum cortisol measurements. Plasma ACTH was increased in all patients at presentation (median = 1050 ng/L, range 68-2630 ng/L, reference range 10-47 ng/L). They were subsequently treated with hydrocortisone, recruited for the therapeutic clinical trials and ACTH_1-24_–stimulated serum cortisol measurements were repeated 14 to 28 days later (median 26 days), before any other intervention. In 19 of the 20 cases, stimulated serum cortisol dropped substantially over the first few weeks of hydrocortisone treatment, from a median at presentation of 125 nmol/L (range < 23-257) to 39.5 nmol/L (range < 23-265), (*P* < 0.007; Mann-Whitney *U* test) (Fig. 1).

### Established Addison disease

**Figure 2.**
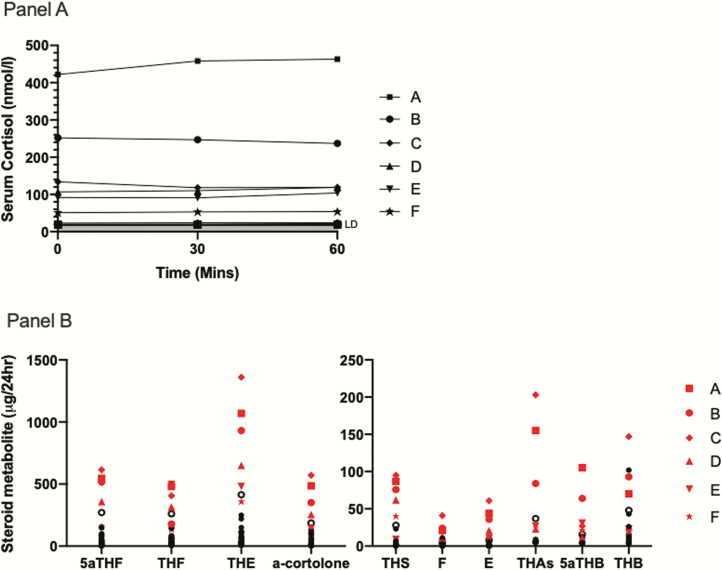
Established Addison disease. Peak serum cortisol following ACTH_1-24_ and corresponding urine glucocorticoid and mineralocorticoid metabolites. (A) Serum cortisol responses at 30 and 60 minutes after ACTH_1-24_. Six participants had serum cortisol concentrations ≥ 30 nmol/L and these are labeled as patients A-F (highest to lowest cortisol) in subsequent figures. All the remaining 31 patients are overlaid with concentrations at, or close to, the limit of detection (20-30 nmol/L). (B) Urine glucocorticoid and mineralocorticoid metabolites that were most correlated with serum cortisol (Table 2), with patients A through F highlighted in red. One participant (subject 27, Table 1) who had a peak serum cortisol of 22 nmol/L nevertheless had consistently detectable urine glucocorticoid and mineralocorticoid metabolites and is shown as an open circle. Abbreviated metabolite names are shown in full in Table 2. Individual participant correlations between peak serum cortisol and the most associated urine glucocorticoid and mineralocorticoid metabolites are shown in Figure 3.

Thirty-seven patients (age range 17-75 years, 26 females) with established Addison disease from between 7 months and 44 years duration were studied (Table 1). Thirty-six patients were confirmed to have primary adrenal insufficiency by an elevated plasma ACTH at baseline following steroid withdrawal (median = 789 ng/L, range 62- >1250 ng/L, reference range 10-47 ng/L). One subject had plasma ACTH in the reference range (19 ng/L) in the baseline sample; a woman with a personal history of autoimmune thyroid disease and a sibling with autoimmune Addison disease and pernicious anaemia, who was taking multiple nonendocrine medications. There were no adverse events reported during steroid withdrawal. Six participants (16%) had a peak serum cortisol clearly above the 20 nmol/L threshold for detection, whereas the remaining 31 patients showed serum cortisol less than 30 nmol/L throughout (Fig. 2). There were no obvious clinical differences between the 6 participants with higher serum cortisol and the others (23). As expected for individuals with high circulating ACTH concentrations at baseline, there was minimal augmentation of serum cortisol following Synacthen stimulation. LC-MS analysis of the steroid metabolome in the overnight urine collection from these 37 participants showed that those with detectable serum cortisol also had secretion of several other glucocorticoid and mineralocorticoids (Fig. 2B).

**Table 1.** Demographics and Treatment of Established Addison Disease Participants at Study Entry

					Steroid replacement		
Patient ID	Age	Sex	Duration of diagnosis (Years)	Plasma ACTH (ng/L)	Glucocorticoid (mg)	Mineralocorticoid (mcg)	Concurrent Autoimmune Conditions
1^A^	24	F	3	126	HC 10 + 5	50	HT
2^B^	41	F	9	651	Pred 2.5 + 1	100	
3^C^	59	M	11	62	HC 20 + 5	150	
4^D^	67	F	31	1107	HC 10 + 5 + 5	100	
5^E^	61	M	41	188	HC 15 + 10	100	
6^F^	52	F	10	957	HC 7.5 + 5	100	HT
7	51	F	8	429	HC 10 + 5	100	GD
8	34	F	3	769	Pred 3	150	
9	64	F	5	809	HC 10 + 5	100	
10	43	F	33	698	HC 10 + 10	200	
11	58	F	25	>1250	HC 10 + 10	50	T1DM, HT, POF
12	58	F	13	>1250	HC 10 + 5 + 5	50	HT, PA
13	72	M	6	591	HC 10 + 5 + 5	100	
14	75	F	10	**19**	Pred 3.5 + 2.5	150	HT
15	48	F	13	210	HC 10 + 5	50	HT, POF, vitiligo
16	57	F	17	1166	HC 10 + 5 + 2.5	100	T1DM, PA
17	27	F	6	532	Pred 5 + 1	900	T1DM
18	37	M	17	470	HC 10 + 10 + 5	100	T1DM, HT
19	63	F	23	238	HC 10 + 10	150	GD
20	68	F	44	1074	HC 10 + 5 + 5	100	HT
21	55	F	28	983	HC 10 + 5	100	
22	71	F	18	>1250	HC 10 + 5 + 5	150	HT
23	17	M	8	>1250	HC 10 + 5 + 2.5	150	
24	25	F	7	>1250	HC 10 + 5 + 5	100	
25	32	M	7	>1250	HC 10 + 10	100	T1DM
26	17	M	4	>1250	Pred 5	300	T1DM, HT
27	33	F	<1	>1250	HC 15 + 10	100	
28	27	M	7	>1250	HC 15 + 10	100	
29	19	M	5	>1250	HC 10 + 7.5 + 2.5	100	
30	67	F	1	693	HC 10 + 5 + 5	100	
31	74	F	47	104	HC 10 + 10	50	
32	67	M	22	>1250	HC 10 + 5 + 5	150	Vitiligo
33	24	M	8	>1250	HC 10 + 5 + 5	200	
34	69	F	41	296	HC 10 + 5	100	
35	60	F	19	219	HC 10 + 5	100	T1DM, GD, PA
36	60	F	23	367	HC 10	100	T1DM, GD, PA
37	46	F	19	149	HC 10 + 5 + 5	200	T1DM, HT

Age, sex, and duration of diagnosis (in years) are included for all participants. All participants were taking hydrocortisone or prednisolone as glucocorticoid replacement, with 34/37 taking this in split doses across the day. All patients were taking fludrocortisone as mineralocorticoid replacement (once daily; range 50-900 μg daily). 19/37 had 1 or more additional autoimmune disease. The 6 participants with a serum cortisol clearly above the 20 nmol/L threshold for detection are labelled A-F.

GD = Graves’ disease, HC = hydrocortisone, HT = hypothyroidism, PA = pernicious anemia, Pred = prednisolone, POF = premature ovarian failure, T1DM = type 1 diabetes

Interestingly, patient B (Table 1, Fig. 2) previously took part in a study looking to regenerate adrenal function (participant 06 from Gan et al.) (12). Before intervention in 2012, her peak serum cortisol was 184 nmol/L, and when measured in 2016 for this study it was 252 nmol/L, suggesting that her residual adrenal function was static and durable between the 4 and 8 years postdiagnosis interval that we have been able to observe.

**Figure 3.**
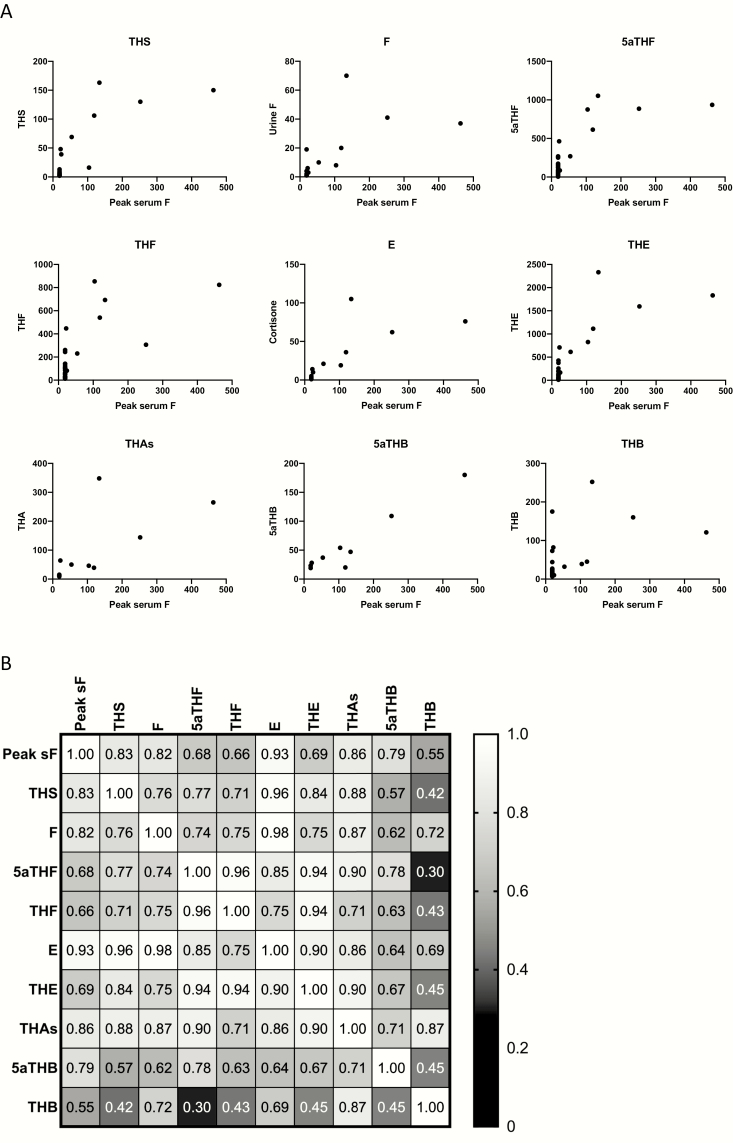
Comparison of peak serum cortisol and urine steroid metabolome. (A) Peak serum cortisol (F) plotted against selected urinary corticosteroid metabolites. (B) Correlation heatmap (Spearman ρ) for serum cortisol and selected urine glucocorticoids and mineralocorticoids. The Spearman’s rank correlation coefficient (ρ) between each pair of urine or serum glucocorticoid or mineralcorticoids is shown in the intersecting square, along with a shaded background indicating the strength of the correlation. Urine glucocorticoid metabolites were more closely associated with peak serum cortisol than mineralocorticoid metabolites. Abbreviated metabolite names are shown in full in Table 2.

All patients with serum cortisol > 30 nmol/L had detectable urine glucocorticoid and mineralocorticoid precursors (Fig. 2). Table 2 shows the correlation of each urine steroid measured with the peak serum cortisol concentration during Synacthen testing. Urine glucocorticoid precursor metabolites were most highly correlated with serum cortisol concentrations, with urine mineralocorticoids including dehydrocorticosterone and corticosterone metabolites being less tightly associated, and adrenal androgens showing the least association. Correlation analysis between the selected urine metabolites and peak serum cortisol is shown in Fig. 3. As expected, several urinary glucocorticoid precursors and metabolites and mineralocorticoids were associated with peak serum cortisol, with tetrahydro-11-deoxycortisol as the most significantly correlated (Table 2, Fig. 3). Interestingly, 1 female participant who had negligible concentrations of serum cortisol post-ACTH_1-24_ stimulation (peak 22 nmol/L) had comparable levels of urine glucocorticoid precursors to several individuals with easily detectable serum cortisol, suggesting that urine steroid analysis may represent a more sensitive indicator of residual steroidogenesis (shown as an open circle in Fig. 2B). This 33-year-old woman was sampled 7 months after diagnosis with AAD; her initial presentation was sepsis and an adrenal crisis. She reported symptoms of fatigue and increasing back pain and had lost 12 kg in weight over the 4 years before presentation.

**Table 2.** Spearman’s ρ Correlation of Peak Post-ACTH_1-24_ Serum Cortisol vs Urine Steroid Metabolome

Precursor/Metabolite	Spearman’s ρ	*P* Value
Peak serum F = cortisol	1	(referent)
PD = pregnanediol	0.423	0.011
17HP = 17-hydroxyprogesterone	0.144	0.475
PT = pregnanetriol	0.339	0.046
PTONE = pregnanetriolone	0.644	0.037
**THS = tetrahydro-11-deoxycortisol**	**0.833**	**4.272e-007**
F = cortisol	0.825	0.001
18OHF = 18-hydroxycortisol	0.963	0.002
**5aTHF = 5a-tetrahydrocortisol**	**0.682**	**4.727e-006**
**THF = tetrahydrocortisol**	**0.658**	**1.730e-005**
a-cortol	0.456	0.015
b-cortol	0.449	0.005
11bOHEt = 11b-hydroxyetiocholanolone	0.426	0.011
**E = cortisone**	**0.934**	**8.209e-007**
**THE = tetrahydrocortisone**	**0.687**	**2.688e-006**
**a-cortolone**	**0.659**	**9.261e-006**
b-cortolone	0.538	0.001
THAs = tetrahydro-11-dehydrocorticosterone	0.858	0.001
5aTHB 5a-tetrahydrocorticosterone	0.787	0.016
THB = tetrahydrocorticosterone	0.546	0.002
THAldo = tetrahydroaldosterone	0.496	0.077
Andros = androsterone	0.224	0.203
Etio = etiocholanolone	0.375	0.024

Correlation between peak stimulated serum cortisol and urinary steroid metabolites using a Spearman rank test. THS, 5aTHF, THF, E, THE and a-cortolone (highlighted in bold) are cortisol, cortisone and 11-deoxycortisol metabolites and are most significantly correlated with peak serum cortisol concentrations.

## Discussion

AAD has a long latency before presentation in most patients (5-7) and understanding the natural history once steroid replacement treatment has started is an important goal if we are to intervene to improve adrenal function in this chronic condition. The initial part of this study documents for the first time that serum cortisol concentrations drop rapidly within 4 weeks following diagnosis and conventional treatment of AAD. The most plausible explanation for this is that the ACTH drive, which maximally stimulates the adrenal cortex before diagnosis, is reduced by the institution of regular daily glucocorticoid medication under physiological feedback. Thus, before diagnosis, patients with AAD have constantly high circulating ACTH concentrations; these serve to stimulate proliferation and repopulation of adrenocortical cells that are being destroyed by immune-mediated attack. Hyperplastic adrenocortical cells were noted many years ago in the adrenals from deceased AAD patients (24, 25). However, once glucocorticoid replacement therapy is started the plasma ACTH drops to low or undetectable levels after the morning dose of hydrocortisone is taken each day, reducing the tropic drive to steroidogenesis and regeneration. This change in ACTH concentration also correlates with the improvement in pigmentation observed in most AAD patients following successful treatment. An alternative explanation is that patients’ adrenal steroidogenic function is on a steep downward trajectory at the time they are diagnosed and that it is declining irrespective of the steroid replacement treatment and changes to ACTH levels. Either way, this phenomenon seems like the opposite of the honeymoon period in type 1 diabetes (26), where there is frequently a transient improvement in islet cell function and insulin secretion in the months following initial insulin therapy.

The second component of our study sought to characterize adrenal function in patients with established autoimmune adrenal failure treated for more than 6 months. The design was distinct from previous studies in that the oral glucocorticoid was withdrawn for 36 to 40 hours before gold-standard testing with Synacthen stimulation, and for 24 hours before the start of collection of urine for steroid metabolomic analysis. This study showed that 6 of 37 (16%) AAD patients had some evidence of residual adrenal function with detectable serum cortisol and urine glucocorticoid and mineralocorticoid metabolites. In addition, at least 1 other participant had clearly detectable urine steroid metabolites in the absence of a significant serum cortisol response. In common with the study of Vulto et al. (17), we found that the cortisol precursor, 11-deoxycortisol, appeared to be a sensitive marker of residual adrenal function; our study measuring urine tetrahydro-11-deoxycortisol and theirs using serum 11-deoxycortisol to reach the same conclusion. In addition, our study found multiple other glucocorticoid metabolites that were strongly associated with residual adrenal function and serum cortisol concentrations, proving that in these stringent, steroid medication-free conditions detectable serum and urine steroid metabolites represent the authentic output of low-level residual adrenocortical function in these patients, rather than a hangover of prior oral medications. In addition to glucocorticoid production, we also found significant mineralocorticoid secretion, which correlated more loosely with serum and urine glucocorticoid levels. In contrast, adrenal androgen production was unhelpful and poorly correlated with either serum glucocorticoid or mineralocorticoid concentrations. This is consistent with our previous observations in a small number of AAD individuals who improved their adrenal steroidogenesis following intervention, in whom no change in adrenal androgens was found despite improvements in serum cortisol and aldosterone concentrations (12, 18). However, there was no standardization for menopausal status or estrogen use in this study, introducing some heterogeneity from gonadal steroid production in this analysis.

Our results indicate that AAD is not a homogeneous condition and that a sizeable minority of patients with AAD may have low-level residual adrenal function. Whether these patients represent a distinct cohort either in terms of their etiopathogenesis, requirement for steroid replacement or resilience to adrenal crisis and other complications remains unknown. However, it seems likely that there is no relationship between time since diagnosis and the presence of residual adrenal function, with several individuals identified with residual function by this study having had AAD treated for more than 10 years. Furthermore, we have been able to study one individual over 4 years and demonstrate that the residual adrenal function was indeed persistent. This would fit with the idea that the adrenocortical stem or progenitor cells remain intact and are spared the autoimmune attack in these individuals (12-15). In contrast to the lack of “honeymoon period” in AAD, it therefore seems that a proportion of patients do have a durable low-level adrenal steroidogenesis, which may be considered parallel to persistent C-peptide positivity indicating low-level insulin secretion in patients with type 1 diabetes. It is clear that future studies that try to augment steroidogenesis in AAD patients should focus primarily on those with detectable residual adrenal function.

The strength of this study lies in the design of testing participants in a medication-free state, which has not been done previously, and in measuring both stimulated serum cortisol concentrations and the urine steroid metabolome. The weaknesses are that all but one patient were studied at just a single time-point, and that although the largest cohort studied in this way to date, it still remains a relatively small cohort of AAD patients.

In conclusion, our study illuminates several novel aspects of the natural history of AAD. First, that patients with newly diagnosed AAD have a rapid decline in steroidogenic capacity once replacement glucocorticoid medication is commenced, presumably owing to reduced adrenal ACTH exposure. We also show that a sizeable minority of AAD patients have durable low-level residual adrenal function even many years from their original diagnosis and institution of replacement therapy. This may be sensitively detected by serum 11-deoxycortisol or its major urinary metabolite tetrahydro-11-deoxycortisol. These observations should help plan future intervention studies that could target specifically patients with residual adrenal function to ameliorate this chronic condition.

## Abbreviations

AAD: autoimmune Addision disease
LC: liquid chromatography
MS/MS: tandem mass spectometry

## Additional Information

***Disclosure Summary:*** S.H.S.P. has consulted for Apitope and received speaker fees from Quidel, Berlin Chemie, and Sanofi. All other authors have no conflict of interest to declare.

***Data Availability:*** All data generated or analyzed during this study are included in this published article or in the data repositories listed in References.
